# Increased Heart Rate during Walk Test Predicts Chronic-Phase Worsening of Renal Function in Patients with Acute Myocardial Infarction and Normal Kidney Function

**DOI:** 10.3390/ijerph16234785

**Published:** 2019-11-29

**Authors:** Asami Ogura, Kazuhiro P. Izawa, Hideto Tawa, Fumie Kureha, Masaaki Wada, Masashi Kanai, Ikko Kubo, Ryohei Yoshikawa, Yuichi Matsuda

**Affiliations:** 1 Department of Public Health, Graduate School of Health Sciences, Kobe University, Kobe 654-0142, Japan; izawapk@harbor.kobe-u.ac.jp (K.P.I.); gjsjk188@yahoo.co.jp (M.K.); ikko7667@yahoo.co.jp (I.K.); 2 Department of Rehabilitation, Sanda City Hospital, Sanda 669-1311, Japan; wdmsk214@yahoo.co.jp; 3 Cardiovascular Stroke Renal Project (CRP), Kobe 654-0142, Japan; 4 Department of Cardiology, Sanda City Hospital, Sanda 669-1311, Japan; tawa_hideto@hospital.sanda.hyogo.jp (H.T.); kureha_fumie@hospital.sanda.hyogo.jp (F.K.); yoshikawa-ryo@w5.dion.ne.jp (R.Y.); ymatsudamd@hospital.sanda.hyogo.jp (Y.M.)

**Keywords:** acute myocardial infarction, worsening renal function, walk test, heart rate

## Abstract

Chronic-phase worsening renal function (WRF) in patients with acute myocardial infarction (AMI) has been associated with poor prognosis. However, there is no consensus on either the method of prevention or the cause. The aim of this study was to determine factors predictive of chronic-phase WRF from the viewpoint of circulatory dynamics response to exercise during hospitalization of AMI patients without renal dysfunction on admission. We studied 186 consecutively AMI patients who underwent the 200-m walk test. Chronic-phase WRF was defined as a 20% decrease in estimated glomerular filtration rate (eGFR) from baseline to 8–10 months after AMI onset. Heart rate (HR) and systolic blood pressure recorded during the 200-m walk test were evaluated as circulatory dynamics responses. In total, 94 patients were enrolled. Multiple linear regression analysis showed that ΔHR (peak-rest) associated significantly with ΔeGFR (*β =* 0.427, *p* = 0.018). The receiver operating characteristic curve of ΔHR to predict chronic-phase WRF showed an area under the curve of 0.77, with a cut-off value of 22.0 bpm having a 95% sensitivity and 55% specificity. Among circulatory dynamics responses during exercise in the acute phase after AMI, ΔHR was an independent predictor of chronic-phase WRF.

## 1. Introduction

Chronic-phase worsening renal function (WRF) in patients with acute myocardial infarction (AMI) has been associated with major adverse cardiac events and an incidence reported to range from 6.4–33% [1,2,3,4,5,6]. Therefore, countermeasures against chronic-phase WRF are important for the prevention of future cardiovascular events, and thus, it is necessary to clarify the cause of its occurrence. A few studies [1,2,3,4,5,6] have reported various predictive factors of chronic-phase WRF, but there is no consistency on these factors. The presumed causes are differences in the study cohorts, the definition of WRF, and variations in clinical practice between institutions. Nevertheless, previous reports showed that predictors of chronic-phase WRF are associated with pathological conditions and treatment during hospitalization.

In this study, we hypothesized that the pathological conditions causing chronic-phase WRF would appear in the response of circulatory dynamics to exercise in the acute phase of AMI. There are few reports on the relationship between chronic-phase WRF and circulatory dynamics response during exercise in the acute phase of AMI. Furthermore, chronic-phase WRF has been reported to be affected by the presence or absence of chronic kidney disease before the onset of AMI [3,5], so exclusion of patients with chronic kidney disease is necessary to investigate the true association between the pathophysiology of AMI and its treatment and chronic-phase WRF. However, chronic-phase WRF in patients with normal kidney function has not been investigated well [6].

Thus, the purpose of this study was to determine factors predictive of chronic-phase WRF from the viewpoint of circulatory dynamics response during exercise during in hospitalized patients with AMI who did not have renal dysfunction on hospital admission.

## 2. Methods

### 2.1. Study Design and Patients

This was a retrospective, single-center, observational study. From January 2015 to December 2018, 186 patients with AMI who underwent an emergency percutaneous coronary intervention and the 200-m walk test by 2 weeks after the admission in our institution were consecutively enrolled in the study. Patients with an estimated glomerular filtration rate (eGFR) ≥ 60 mL/min/1.73 m^2^ on admission was included. Exclusion criteria included patients with no data on the 200-m walk test or no laboratory data on admission, patients in atrial fibrillation, and patients with no eGFR data recorded for the first 8–10 months after AMI. All patients underwent cardiac rehabilitation according to the Guidelines for Rehabilitation in Patients with Cardiovascular Disease (JCS 2012) [7].

Patients’ characteristics and clinical parameters including age, sex, body mass index, peak creatine phosphokinase, ST-elevation myocardial infarction, left ventricular ejection fraction, contrast volume, pain to balloon time, medical history, laboratory results on admission (blood urea nitrogen/creatinine, brain natriuretic peptide concentration, serum C-reactive protein, hemoglobin, hemoglobin A1c), eGFR between 8–10 months after AMI, medicine taken at the time of the walk test and at discharge, and the results of the 200-m walk test were obtained from the electronic medical records by two physical therapists.

This study complied with the Declaration of Helsinki with respect to investigation in humans and was approved by the Ethics Committee of Sanda City Hospital (approval no. 2019003). Written informed consent was obtained from each patient.

### 2.2. Definitions

#### 2.2.1. AMI

The diagnosis of AMI was confirmed by electrocardiogram findings and elevation of cardiac enzymes. Pain to balloon time was defined as the time from symptom (usually chest pain or discomfort) onset to reperfusion [8].

#### 2.2.2. Renal Function

The Modification of Diet in Renal Disease (MDRD) equation is one of the most commonly used equations for calculating estimated glomerular filtration rate (eGFR) [9]. In this study, eGFR was evaluated with the Japanese version of the equation (JMDRD): eGFR = 194 × (serum creatinine) − 1.094 × age − 0.287 (× 0.739 if female) [10]. eGFR was extracted from the electronic medical records by two physical therapists at the time of hospital admission before percutaneous coronary intervention and at 8–10 months after percutaneous coronary intervention. Patients with eGFR < 60 at admission were excluded from study as chronic kidney disease.

#### 2.2.3. Chronic-Phase WRF

This was defined as a ≥20% decrease in eGFR [11] from admission to 8–10 months after AMI onset. We divided the patients into two groups, the chronic-phase WRF group and the non- chronic-phase WRF group, according to previous studies [1,2].

#### 2.2.4. Walk Test

The 200-m walk test was performed according to the Guidelines for Rehabilitation in Patients with Cardiovascular Disease (JCS 2012) [7]. All tests were conducted between 2 pm and 3 pm. Patients were instructed to lie on a bed for 30 min before the walk test. The patients were told to walk a straight corridor of 50 m one way at their usual walking speed. The variables chosen for monitoring were heart rate (HR) and systolic blood pressure (SBP) as indicators of circulatory dynamics response. We measured the resting (Rest) and peak (Peak) HRs and ΔHR (Peak-Rest). Likewise for SBP, we measured the resting (Rest) and post-test (Post) SBP and ΔSBP (Post-Rest). HR (Rest) was measured from a 12-lead electrocardiogram recorded with the patient in the supine position, and HR (Peak) was measured from lead II of a 3-lead electrocardiogram recorded using the limb lead method during the walk test. SBP (Rest) and SBP (Post) were also measured with the patient in the supine position. These measurements were obtained by one physical therapist and one nurse.

### 2.3. Statistics

Data are expressed as mean values ± standard deviation (SD) or median and interquartile range for continuous variables, as appropriate. Normality of distribution was verified using the Shapiro-Wilk test. Continuous variables were compared by Student *t*-test or Mann-Whitney test, and categorical data were compared by Fisher’s exact test. A multiple linear regression analysis was used to evaluate the contribution of HR and SBP for the absolute change in eGFR from that on admission to that at 8–10 months (ΔeGFR). The confounding factors were selected the variables associated (*p* < 0.05) with ΔeGFR in a simple linear regression analysis. For continuous variables among the predictors extracted by multivariate analysis, cut-off values to predict WRF were defined maximum (sensitivity + specificity), and calculated using receiver operating characteristic (ROC) curves. Validity of the test was assessed by calculating area under curve (AUC). A *p* value of < 0.05 was considered to indicate statistical significance. All statistical analyses were performed with EZR (Saitama Medical Center, Jichi Medical University, Saitama, Japan), which is a graphical user interface for R (The R Foundation for Statistical Computing, Vienna, Austria). More precisely, it is a modified version of R commander designed to add statistical functions frequently used in biostatistics.

## 3. Results

**Figure 1:**
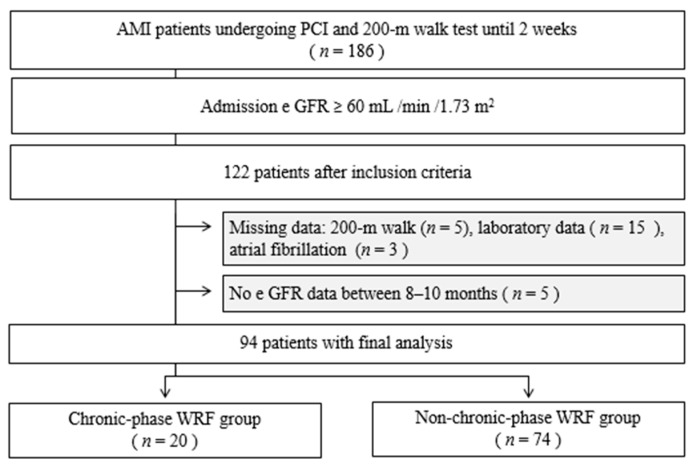
Flow of patients through the study.

In total, 94 patients (mean age 63.6 ± 11.0 years; range, 38 to 85 years) were enrolled in the final analysis. The chronic-phase WRF group comprised 20 patients (21.3%). Figure 1 shows the flowchart of the study population selection.

Patient characteristics are presented in Table 1. Patients in the chronic-phase WRF group included more females than males and had longer pain to balloon time and higher BUN/CRE and BNP values than those in the non-chronic-phase WRF group. eGFR was significantly higher in the patients in the chronic-phase WRF group than in those in the Non-chronic-phase WRF group at admission. However, this group showed a decrease in eGFR of 24.4 mL/min/1.73 m^2^ after 8–10 months, whereas eGFR in the non-chronic-phase WRF group decreased only by 4.1 mL/min/1.73 m^2^. In the 200-m walk test, ΔHR was significantly higher in the chronic-phase WRF group than those in the non-chronic-phase WRF group.

Multiple linear regression analysis showed that ΔeGFR associated significantly with female sex (*β* = 9.631, *p* = 0.004), ΔHR (*β* = 0.427, *p* = 0.018), admission eGFR (*β* = 0.418, *p* < 0.001), and pain to balloon time (*β* = 0.016, *p* = 0.004) (Table 2). Moreover, the significance of the results is also proved, because the values of t-statistics are also significant and are greater than the cutoff value of 1.96 [12].

**Figure 2:**
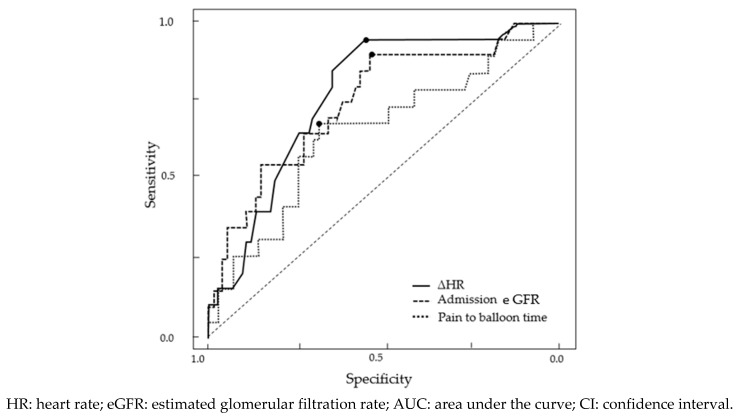
Receiver operating characteristic curve for WRF.

ROC curve analysis was performed to reveal cut-off values for ΔHR, admission eGFR, and pain to balloon time for prediction of WRF (Figure 2). The AUC for detection of WRF was 0.77 (95% confidence interval (CI): 0.67–0.88) of ΔHR. Using a ΔHR cut-off value of 22.0 bpm, the sensitivity and specificity for the prediction of WRF were 95% and 55%, respectively. The AUC for the prediction of WRF was 0.75 (95% CI: 0.63–0.88) of the admission eGFR. Using an admission eGFR cut-off value of 72.3 mL/min/1.73 m^2^, the sensitivity and specificity for the prediction of WRF were 90% and 54%, respectively. The AUC for prediction of WRF was 0.66 (95% CI: 0.51–0.88) of pain to balloon time. Using a pain to balloon time cut-off value of 241.0 min, the sensitivity and specificity for detection of WRF were 68% and 69%, respectively. The result with the highest sensitivity was ΔHR.

## 4. Discussion

In this study, chronic-phase WRF occurred after percutaneous coronary intervention for AMI in 21.3% of patients who had normal kidney function on hospital admission. In previous reports, the prevalence of chronic-phase WRF varied from 6.4 to 33% [1,2,3,4,5,6]. These wide ranges regarding the incidence of chronic-phase deterioration of renal function reported previously may be explained by differences in the study cohorts, the definition of WRF, and the variations in clinical practice between institutions [4]. Nevertheless, we observed that even in AMI patients with normal kidney function, deterioration of renal function was common.

### 4.1. Risk Factors for Chronic-Phase WRF

We showed that female sex, pain to balloon time, admission eGFR, and ΔHR at 200-m walk test were independent risk factors for chronic-phase WRF. These factors are different from those in previous reports [1,2,3,4,5,6], which reported various factors such as old age, low body mass index, baseline renal dysfunction, hemoglobin, troponin levels, diabetes mellitus, amount of contrast, nephrotoxic drugs, and in-hospital initial hemodynamic instability. Almost all of the previous reports included chronic kidney disease patients on the baseline [1,2,3,4,5], who are susceptible to contrast media, drugs, and hemodynamic instability [13]. Although we think that excluding the effect of chronic kidney disease is very beneficial when considering the mechanism of WRF, even in a report on patients with AMI and normal kidney function, the predictors were different from those of the present study [6]. This was thought to be due to a difference in the definition of WRF and the variables used. Even in the guidelines [11], there are several definitions of WRF, which is a factor that complicates this discussion. In any case, when considered the factors involved in chronic-phase WRF, it is necessary to confirm patient background, the definition of WRF used, and the variables.

### 4.2. Association between ΔHR and Chronic-Phase WRF

To our knowledge, this is the first study to verify that the circulatory dynamics response during exercise in the acute phase of AMI can predict chronic-phase WRF. Among the circulatory dynamics responses occurring during exercise, it became clear that ΔHR is an independent predictor of chronic-phase WRF. Neither HR at rest nor peak HR nor SBP was associated with the occurrence of chronic-phase WRF. HR is a powerful tool in the assessment of the autonomic nervous system [14]. HR during exercise is regulated by the autonomic nervous system, which is increased by parasympathetic withdrawal and sympathetic hyperactivity [15]. It is reported that parasympathetic withdrawal and sympathetic hyperactivity occur even in AMI patients without complications [16], and these were considered to be factors involved in the increase of ΔHR in the present study. In addition, sympathetic hyperactivity is also inferred from the cut-off value of ΔHR (22 bpm) that predicts chronic-phase WRF in the present study. In a previous report [17], ΔHR during a 6-min walk test performed in the acute phase of AMI was 25 bpm. Also, according to a cardiopulmonary exercise test performed one week after AMI onset, ΔHR up to the anaerobic threshold was 23 bpm [18]. Although there is a problem with comparing HRs in different types of exercise, HR in the chronic-phase WRF patients during the 200-m walk test in the acute phase of AMI increased to a level comparable to that in the 6-min walk test, and it may have exceeded anaerobic threshold. Sympathetic hyperactivity further increases above the anaerobic threshold [19]. The 200-m walk test is likely to reflect exercise patterns in daily life [20], and it suggests that chronic-phase WRF patients are sympathetic predominant in daily life.

### 4.3. Sympathetic Hyperactivity and Chronic-Phase WRF

Because sympathetic hyperactivity after AMI has been reported to cause deterioration of renal function [21,22], daily sympathetic hyperactivity may have caused chronic-phase WRF in the study patients. However, because not all patients with AMI experience a deterioration of renal function, we think that such deterioration may occur if sympathetic hyperactivity becomes more severe as a result of certain factors after AMI onset. In this respect, it is interesting that female sex and pain to balloon time were also extracted as predictors of chronic-phase WRF in the present study. Enhancement of the sympathetic nervous system after AMI has been shown to occur more often in females than in males [23], and it was reported that sympathetic hyperactivity in AMI patients is associated with ischemic time [24]. It can be presumed that female sex and long ischemic times may be related to increased sympathetic nervous stimulation that can lead to deterioration of renal function in the chronic phase. It is possible that not only ΔHR during the walk test but also other risk factors are related to sympathetic hyperactivity, lending support to the cause of increased HR being sympathetic hyperactivity.

### 4.4. Sympathetic Hyperactivity and Other Parameters of the 200-m Walk Test

If the sympathetic nervous system is enhanced, it can be predicted that the resting HR will be high, but in the present study, there was no significant difference in the resting HR between the two groups. There is a previous report of this phenomenon [25], and even if there is no difference in parasympathetic nerve activity at rest, the attenuation of parasympathetic nerve activity during exercise may increase. The present study showed that SBP had no relevance. It was reported that ΔSBP was 4 mm Hg even in the 6-min walk test targeting AMI patients [17]. We considered that the light exercise load applied in the present study would not be enough to raise the SBP.

### 4.5. Usefulness of the 200-m Walk Test

Although the 200-m walk test is a self-selected (comfortable) speed test in which a quantitative load is not measured, it is indicated in the Guidelines for Rehabilitation in Patients with Cardiovascular Disease as a load test for AMI patients [7], and several studies have used it as an evaluation item [26,27]. The 50-m and 100-m walk tests have also been reported as similar self-selected (comfortable) speed tests for patients with cardiovascular disease [20]. Their advantage is that they are suitable for obtaining a response during daily exercise rather than forcing the patient to perform an unusual exercise style such as walking on a treadmill. Although, clinically significant changes in, and the reliability of, these self-selected speed tests are still the subject of debate, the fact that factors related to the future deterioration of renal function can be extracted from the 200-m walk test may help to indicate the clinical significance of this test.

### 4.6. Clinical Implication and Future Research

Our findings suggested that the pathological condition causing chronic-phase WRF appeared in the circulatory dynamics response during exercise in the acute phase of AMI. Sympathetic nerve activity was inferred as the pathological condition causing WRF, and it is presumed that it appeared as HR rises easily during walking. ΔHR measured during a walk test may be a non-invasive and convenient method of predicting patients with chronic-phase WRF. The ability to easily predict chronic-phase WRF contributes to increasing opportunities to undertake countermeasures. Furthermore, exercise therapy has been reported to suppress sympathetic hyperactivity [28]. If sympathetic hyperactivity is the cause of WRF, exercise therapy may possibly prevent chronic-phase WRF.

Further research is needed to quantitatively evaluate sympathetic activity and confirm its relationship with WRF. Furthermore, the expectation that chronic kidney disease patients would follow a worse course than patients with normal kidney function should also be investigated.

### 4.7. Limitations

There are several limitations associated with our analyses. First, this was a single-center study with a small number of patients that might create a risk of patient selection bias. Second, the value of serum creatinine on admission may not reflect true baseline renal function as we also included patients with heart failure and cardiogenic shock. Third, the 200-m walk test was not a quantitative load. Finally, we could not determine the presence or absence of acute kidney injury and other episodes that can cause deterioration of renal function after discharge.

## 5. Conclusions

Chronic-phase WRF in patients with AMI and normal kidney function is not common complication. In these patients, increased HR during a 200-m walk test was an independent predictor for chronic-phase WRF. Our hypothesis that the pathological condition causing chronic-phase WRF appears in the circulatory dynamic response during exercise in the acute phase of AMI was proved. The present findings suggested that sympathetic hyperactivity may increase HR during a walk test and cause deterioration of renal function in the chronic phase after AMI.

## Figures and Tables

**Table 1:** Clinical characteristics of the patients.

	Chronic-Phase WRF *n* = 20	Non-Chronic-Phase WRF *n* = 74	*p* Value
Age, years	66.3 ± 11.0	62.9 ± 11.0	0.225
Female, *n* (%)	7 (35)	7 (9.5)	0.009
Body mass index, kg/m^2^	22.6 (20.9–23.9)	23.8 (21.9–25.9)	0.116
Peak CPK, IU/L	1822 (920.0–3490.3)	1917.0 (843.8–3142.0)	0.989
STEMI, *n* (%)	15 (75.0)	59 (79.7)	0.759
LVEF, (%)	53.1 ± 9.4	57.3 ± 11.9	0.149
Contrast volume, mL	150 (133.8–190.0)	140 (115.0–170.0)	0.425
Pain to balloon time, min	285 (164.0–497.5)	179.0 (131.0–296.3)	0.039
Medical history			
Previous MI, *n* (%)	0 (0)	6 (8.1)	0.336
Hypertension, *n* (%)	13 (65.0)	45 (60.8)	0.800
Diabetes mellitus, *n* (%)	5 (25.0)	27 (36.5)	0.430
200-m walk test			
Rest HR, bpm	63.1 ± 10.2	64.5 ± 8.7	0.522
Peak HR, bpm	91.8 ± 12.9	86.4 ± 10.6	0.057
ΔHR (peak-rest), bpm	28.7 ± 7.3	21.8 ± 7.0	<0.001
Rest SBP, mmHg	108.5 (100–114.5)	111.0 (102–119.5)	0.567
Post SBP, mmHg	113.5 (101.8–124.5)	118.0 (107.3–123.0)	0.563
ΔSBP (post-rest), mmHg	6.1 ± 11.4	5.9 ± 11.3	0.953
eGFR, mL/min/1.73 m^2^			
Admission	81.5 (74.5–96.0)	71.3 (64.7–78.9)	<0.001
8–10 months	60.2 ± 13.3	69.9 ± 11.6	0.002
Δ (admission–8–10 months)	24.4 (20.1–29.6)	4.1 (−2.2–8.6)	<0.001
Admission laboratory findings			
BUN/CRE	21.0 (18.1–27.9)	19.0 (16.2–21.3)	0.075
BNP, pg/mL	65.2 (22.3–116.8)	20.7 (6.9–43.6)	0.001
Serum CRP, nmol/L	0.46 (0.06–2.34)	0.14 (0.05–0.68)	0.322
Hemoglobin, g/dL	14.3 ± 1.8	14.9 ± 1.7	0.159
HbA1c, %	5.9 (5.7–6.4)	6.1 (5.8–6.9)	0.119
Medications at 200-m walk			
Beta blockers, *n* (%)	11 (55.0)	43 (58.9)	0.802
Medications at discharge			
Beta blocker, *n* (%)	13 (65.0)	40 (57.1)	0.612
ACE-I, *n* (%)	5 (25.0)	19 (26.0)	1
ARB, *n* (%)	6 (30.0)	25 (34.2)	0.795
CCB, *n* (%)	3 (15.0)	12 (16.4)	1
Diuretic, *n* (%)	6 (30.0)	8 (11.0)	0.070

WRF: worsening renal function; CPK, creatine phosphokinase; STEMI, ST segment elevation myocardial infarction; LVEF, left ventricular ejection fraction; MI, myocardial infarction; HR, heart rate; SBP, systolic blood pressure; eGFR, estimated glomerular filtration rate; BUN/CRE, blood urea nitrogen/creatinine; BNP, brain natriuretic peptide; CRP, C-reactive protein; HbA1c, hemoglobin A1c; ACE-I, angiotensin converting enzyme inhibitor; ARB, angiotensin II receptor blocker; CCB, calcium channel blocker. Values shown are *n* (%), mean ± standard deviation, or median (interquartile range).

**Table 2:** Multiple linear regression models testing predicting ΔeGFR.

	Univariate			Multivariate			
	*β*	95% CI	*p*-Value	*β*	95% CI	t	*p*-Value
Age	0.063	−0.180, 0.308	0.606				
Female	8.542	1.233, 15.852	0.023	9.631	3.190, 16.073	2.975	0.004
Body mass index	−0.375	−1.108, 0.359	0.313				
Peak CPK	<0.001	−0.001, 0.001	0.891				
STEMI	−1.485	−9.787, 6.818	0.723				
LVEF	−0.109	−0.348, 0.131	0.370				
Contrast volume	0.033	−0.025, 0.090	0.267				
Pain to balloon time	0.020	0.007, 0.033	0.004	0.016	0.005, 0.027	2.970	0.004
Medical history							
Previous MI	−10.042	−20.800, 0.711	0.067				
Hypertension	0.871	−4.634, 6.375	0.754				
Diabetes mellitus	−3.522	−9.125, 2.018	0.215				
200-m walk test							
Rest HR	−0.065	−0.363,0.234	0.668				
Peak HR	0.252	0.019, 0.486	0.034	−0.017	−0.245, 0.212	−0.145	0.885
ΔHR (peak-rest)	0.645	0.317, 0.973	<0.001	0.427	0.076, 0.777	2.419	0.018
Rest SBP	−0.022	−0.165, 0.208	0.817				
Post SBP	0.049	−0.111, 0.208	0.547				
ΔSBP (post-rest)	0.073	−0.156, 0.312	0.545				
Admission laboratory findings							
eGFR	0.507	0.351, 0.664	<0.001	0.418	0.265, 0.571	5.415	<0.001
BUN/CRE	0.632	0.125, 1.138	0.015	0.009	−0.431, 0.449	0.042	0.967
BNP	0.005	−0.019, 0.029	0.665				
CRP	1.278	−0.407, 2.963	0.135				
Hemoglobin	−1.046	−2.609, 0.517	0.187				
HbA1c	−0.831	−3.508, 1.847	0.539				
*R* ^2^							0.439

eGFR, estimated glomerular filtration rate; CI: confidence interval; CPK, creatine phosphokinase; STEMI, ST segment elevation myocardial infarction; LVEF, left ventricular ejection fraction; MI, myocardial infarction; HR, heart rate; SBP, systolic blood pressure; BUN/Cre, blood urea nitrogen/creatinine; BNP, brain natriuretic peptide; CRP, C-reactive protein; HbA1c, hemoglobin A1c.
